# Magnifying endoscopy with narrow-band imaging is useful in differentiating gastric cancer from matched adenoma in white light imaging

**DOI:** 10.1038/s41598-022-12315-0

**Published:** 2022-05-19

**Authors:** Naoki Tamura, Yoshiki Sakaguchi, Wakiko Furutani, Maki Matsui, Sayaka Nagao, Nobuyuki Sakuma, Kazushi Fukagawa, Yuko Miura, Hiroya Mizutani, Daisuke Ohki, Yosuke Kataoka, Itaru Saito, Masayoshi Ono, Chihiro Minatsuki, Yosuke Tsuji, Satoshi Ono, Shinya Kodashima, Hiroyuki Abe, Tetsuo Ushiku, Nobutake Yamamichi, Kazuhiko Koike, Mitsuhiro Fujishiro

**Affiliations:** 1 Department of Gastroenterology, Graduate School of Medicine, The University of Tokyo Hospital, 7-3-1, Hongo, Tokyo, 113-8655 Japan; 2 Department of Gastroenterology, Hokkaido University Hospital, Kita14, Nishi5, Kita-Ku, Sapporo, Hokkaido 060-8648 Japan; 3 Department of Pathology, The University of Tokyo Hospital, 7-3-1, Hongo, Tokyo, 113-8655 Japan

**Keywords:** Gastric cancer, Oesophagogastroscopy

## Abstract

This study assessed the effect of magnifying endoscopy with narrow-band imaging (M-NBI) on the endoscopic differential diagnosis between intramucosal gastric carcinomas and adenomas with matched characteristics. Associations between magnified endoscopic findings and pathological high-grade cellular and architectural atypia were also investigated. In total, the records of 50 adenomas and 50 intramucosal well-differentiated adenocarcinomas matched by tumor size (≥ 20 mm or < 20 mm), shape (depression or non-depression), and color (red or non-red) were extracted. Fourteen endoscopists diagnosed adenoma or cancer in the 100 cases with conventional white light imaging (C-WLI), then did the same with C-WLI + M-NBI.The cancer diagnostic sensitivity, specificity, and accuracy were assessed. The sensitivity of C-WLI + M-NBI for cancer diagnosis was 79.9% compared to 71.6% with C-WLI (p < 0.001). There were no significant differences in specificity (40.1% vs. 36.3%, p = 0.296) and accuracy (55.9% vs. 58.1%, p = 0.163). High-grade cytological or architectural atypia was diagnosed more often with irregular microvascular pattern (IMVP) or microsurface pattern (IMSP), respectively, than the low-grade forms. In conclusion, IMVP and IMSP correlate with high-grade cytological and architectural atypia. M-NBI is useful in differentiating intramucosal carcinoma from adenoma and can reduce underdiagnosis of cancer.

## Introduction

Gastric cancer is the third leading cause of cancer death worldwide^1^. However, the 5-year relative survival rate of stage IA early gastric cancer is higher than 95%. Early gastric cancer can often be curatively treated with endoscopic resection. As endoscopic therapy is a stomach-preserving technique and minimally invasive, a higher standard of quality of life (QOL) can be preserved with endoscopic treatment compared to surgical treatment^2^. On the other hand, the 5-year relative survival rate of advanced gastric cancer with distal metastasis is lower than 10%^3,4^. Therefore, diagnosing gastric cancer at an early stage is very important for both improvements of prognosis and preservation of gastric function.

In recent years, the detection rate of early gastric cancer has increased owing to the progress of endoscopic devices and diagnostic methods^5^. Early gastric cancer is often difficult to differentiate endoscopically from gastric adenoma, which is a benign epithelial tumor. In addition, lesions diagnosed as gastric adenoma by biopsy often result in a final diagnosis of cancer after endoscopic resection. There are several reports on the diagnostic accuracy of gastric adenoma and gastric cancer with conventional white light imaging (C-WLI). Kato et al. reported on endoscopic submucosal dissection (ESD) for 468 cases of gastrointestinal noninvasive neoplasia with preoperative Vienna classification category 3 or 4.1. They showed that the underdiagnosis rate after ESD was 44%, and that cancer was larger and more depressed than adenoma^6^. Cho et al. reported that in 236 cases of low-grade dysplasia (LGD)/adenoma on preoperative biopsy, 33.9% were high-grade dysplasia (HGD)/carcinoma after endoscopic resection and showed that the HGD/carcinoma are more depressed, red, and larger than LGD/adenoma^7^. In addition, Kim et al. reported that in 285 cases of LGD on preoperative biopsy, 16.1% were HGD/carcinoma, and showed that more than 2 cm, erythema, and depression were associated with upgraded histology after resection^8^. In this way, findings such as size, depression, and redness are known to be effective in differentiating gastric cancer from gastric adenoma with C-WLI. However, in the 285 cases Kim et al. mentioned above, regarding the findings for diagnosing cancer, the sensitivity and specificity for a size 2 cm or more were 37% and 87%, respectively; for redness, the sensitivity and specificity were 37% and 94%; and for depression, the sensitivity and specificity were 15% and 98%, respectively. Thus, diagnosis with C-WLI and preoperative biopsy have limitations in differentiating gastric cancer from adenoma. There is a risk of underdiagnosing cancer with diagnoses using only C-WLI or preoperative biopsy.

In addition to C-WLI, since the diagnostic algorithm for early gastric cancer using M-NBI (MESDA-G) was published in 2016, diagnosis of early gastric cancer with M-NBI is used widely in Japan^9^. In this algorithm, gastric cancer is diagnosed when a demarcation line is present and an irregular microvascular pattern (IMVP) or irregular microsurface pattern (IMSP) can be confirmed with M-NBI. Although there are a few reports on the efficacy of M-NBI for differentiation between gastric cancer and gastric adenoma, it is not clear how effective M-NBI is for lesions that are difficult to differentiate with C-WLI^10–12^. Therefore, in this study, we investigated the effect of M-NBI in differentiating gastric cancer and adenoma matched by tumor size, shape, and color.

In pathological diagnosis, there is a difference in the diagnosis of cancer and dysplasia/adenoma between Europe and the United States and Japan. In Europe and the U.S., cancer is diagnosed by the disruption of the basal membrane in conjunction with the spread of cancer cells into the lamina propria. In contrast, in Japan, the diagnosis is based on cellular atypia and architectural atypia, irrespective of evidence of stromal invasion^13–15^. In a report on the Vienna classification, Western pathologists and Japanese pathologists compared 35 gastric tumors divided into five categories: reactive epithelium, low-grade adenoma/dysplasia, high-grade adenoma/dysplasia, suspected carcinoma, and definite carcinoma. Of the 29 cases diagnosed as definite cancer by Japanese pathologists, 3 were low-grade adenoma/dysplasia, 12 were high-grade adenoma/dysplasia and 10 were definite cancer by Western pathologists^14^. These results show Japanese pathologists diagnose cancer differently than Western pathologists. M-NBI may be able to observe the fine structure of the lesion in more detail than C-WLI. If cellular and architectural atypia can be detected endoscopically, it may be possible to diagnose cancer at an earlier stage.

The association between pathological cytological and architectural atypia with IMVP and IMSP in M-NBI findings was also investigated in this study.

## Results

### Patient characteristics

In the pre-matching patient characteristics, the frequencies of depression (56% vs 22%, p < 0.001) and red color (18% vs 1%, p < 0.001) were significantly higher in the cancer group than in the adenoma group. Tumor size was larger in the cancer group than in the adenoma group (9.9 mm vs 13 mm, p = 0.019), but there was no significant difference between the proportions of ≥ 20 mm and < 20 mm (Table 1).

**Table 1:** Pre-matching patient characteristics.

	Adenoma (n = 68)	Cancer (n = 623)	p value
Male/Female (M%)	47/21(69%)	487/136 (78%)	0.091
Age, years, mean ± SD	69.9 ± 9.0	71.9 ± 8.8	0.074
**Lesion site**			
U/M/L/RS	1/41/25/1	87/263/246/27	**0.004**
Ant/Post/Less/Gre	12/14/22/20	114/126/258/125	0.282
Size, mm, mean ± SD	9.9 ± 5.7	13.0 ± 9.7	**0.019**
≥ 20 mm/< 20 mm (≥ 20 mm%)	6/62 (9%)	114/509 (18%)	0.050
Depression +/- (+ %)	15/53 (22%)	351/272 (56%)	**< 0.001**
Red +/- (+ %)	1/67 (1%)	115/508 (18%)	**< 0.001**
*H. pylori* test +/- (+ %)	28/40 (41%)	189/434 (30%)	0.068
**Atrophy**			
Open/Close/No atrophy	1/0/67	575/46/2	0.163

Significant values are in bold.

*U* upper, *M* middle, *L* lower, *RS* remnant stomach.

*Ant* Anterior wall, *Post* posterior wall, *Less* lesser curvature, *Gre* greater curvature.

In the post-matching patient characteristics, the patients in the cancer group were significantly older than patients with adenoma (73.0 years old vs. 68.1 years old, p = 0.003). There were no significant differences in the sex or location of the lesion, *Helicobacter pylori* (*H. pylori*) infection status, and mucosal atrophy between the cancer and adenoma groups (Table 2).

**Table 2:** Post-matching patient characteristics.

	Adenoma (n = 50)	Cancer (n = 50)	p value
Male/Female (M%)	35/15 (70%)	39/11 (78%)	0.362
Age, years, mean ± SD	68.1 ± 8.6	73.0 ± 7.3	**0.003**
**Lesion site**			
U/M/L/RS	1/27/22/0	5/27/16/2	0.132
Ant/Post/Less/Gre	7/7/20/16	8/8/24/10	0.597
Size ≥ 20 mm/< 20 mm (≥ 20 mm%)	6/44 (12%)	6/44 (12%)	1.000
Depression +/- (+ %)	11/39 (22%)	11/39 (22%)	1.000
Red +/- (+ %)	1/49 (2%)	1/49 (2%)	1.000
*H. pylori* test +/- (+ %)	23/27 (46%)	17/33 (34%)	0.221
**Atrophy**			
Open/Close (open%)	50/0 (100%)	47/3 (94%)	0.242

Significant values are in bold.

*U* upper, *M* middle, *L* lower, *RS* remnant stomach.

*Ant* Anterior wall, *Post* posterior wall, *Less* lesser curvature, *Gre* greater curvature.

### Endoscopic diagnosis

In pre-matching 691 cases (68 adenomas and 623 tub 1), when size ≥ 20 mm was diagnosed as cancer, the sensitivity, specificity, and accuracy were 18%, 91%, and 25%. Similarly, when depression was diagnosed as cancer, the sensitivity, specificity, and accuracy were 56%, 78%, and 58%. When redness was diagnosed as cancer, the sensitivity, specificity, and accuracy were 18%, 99%, and 26%, respectively.

In 100 post-matched cases, the mean values of sensitivity, specificity, accuracy, and the positive predictive value (PPV) and negative predictive value (NPV) with C-WLI were 71.6%, 40.1%, 55.9%, 54.6% and 60.1%, respectively. Similarly, the mean values of sensitivity, specificity, accuracy, and PPV and NPV with C-WLI + M-NBI were 79.9%, 36.3%, 58.1%, 56.0% and 65.2%, respectively (Table 3). The sensitivity of C-WLI and M-NBI was significantly higher than that of C-WLI (79.9% vs. 71.6%, p < 0.001), and NPV was significantly higher than that of C-WLI (65.2% vs. 60.1%, p = 0.025). There were no significant differences in specificity, accuracy, and PPV between C-WLI and C-WLI + M-NBI.

**Table 3:** Diagnostic rate of C-WLI and C-WLI + M-NBI.

	C-WLI (%)	C-WLI + M-NBI (%)	p value
Sensitivity, mean ± SD	71.6 ± 12.1	79.9 ± 10.3	**< 0.001**
Specificity, mean ± SD	40.1 ± 11.9	36.3 ± 15.3	0.296
Accuracy, mean ± SD	55.9 ± 4.5	58.1 ± 4.7	0.163
PPV, mean ± SD	54.6 ± 3.9	56.0 ± 4.0	0.263
NPV, mean ± SD	60.1 ± 10.4	65.2 ± 8.3	**0.025**

Significant values are in bold.

Sensitivity, specificity, accuracy, PPV, NPV represent the mean ± SD of 14 endoscopists.

**Figure 1:**
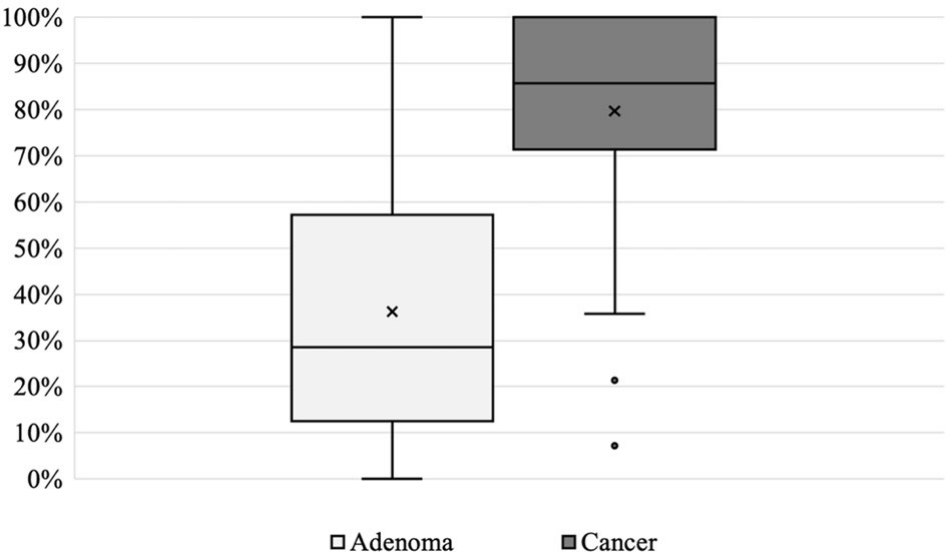
Distribution of accurate diagnosis in 50 gastric adenomas and 50 gastric cancers. The left side axis shows the percentage of endoscopists whose diagnosis with C-WLI + M-NBI was consistent with pathology.

Neither patient factors (age, sex, *H. Pylori* test, mucosal atrophy), lesion factors (site, size, shape, color), or endoscopist factor (years of M-NBI experience) were associated with the accuracy of diagnosis using C-WLI + M-NBI (Table 4). In terms of the inter-endoscopist agreement, Fleiss's kappa was 0.321, a fair agreement. The percentage of accurate diagnosis by the 14 endoscopists for each case varied widely from 0 to 100% (Fig. 1).

**Table 4:** Association between the accuracy of C-WLI + M-NBI and background factors.

	C-WLI + M-NBI accuracy (%)				p value
**Patient factors**					
Male/Female	61		54		0.278
Age < 80 years/ ≥ 80 years	58		64		0.647
*H. pylori* test ±	62		55		0.358
Mucosal atrophy Close/Open	86		58		0.190
**Lesion factors**					
Lesion site					
U/M/L/RS	76	60	54	68	0.543
Ant/Post/Less/Gre	57	51	61	62	0.707
Size ≥ 20 mm/ < 20 mm	53		60		0.371
Depression ±	53		61		0.578
Red ±	57		59		0.941
**Endoscopists factors**					
Years of M-NBI experience ≥ 3 years/< 3 years	60		59		0.443

C-WLI + M-NBI accuracy represents the mean of endoscopic diagnoses consistent with pathological diagnoses.

### Association between M-NBI findings and pathological atypia

After a review of the 100 intramucosal tumors by two expert pathologists, low-grade cellular atypia was found in 78 cases and high-grade cellular atypia in 22 cases. Low-grade architectural atypia occurred in 49 cases and high-grade architectural atypia in 51 cases. The low-grade architectural atypia was less than 50 because of pathological reevaluation.

For high cellular atypia vs low cellular atypia, the ratio of endoscopists diagnosing IMVP was 64% vs. 46%, p = 0.018, with significantly more IMVP in high-grade cellular atypia. The ratio of endoscopists diagnosing IMSP was 66% vs. 54%, p = 0.024, and significantly more IMSP were found in high cellular atypia.

Similarly, for high architectural atypia vs low architectural atypia, the ratio of endoscopists diagnosing IMVP was 59% vs 40%, p = 0.003, and the ratio of endoscopists diagnosing IMSP was 66% vs 47%, p < 0.001, thus IMVP and IMSP were also significantly more common in high architectural atypia (Table 5). These results suggest that pathological high-grade cellular atypia and high-grade architectural atypia are associated with IMVP and IMSP in M-NBI, respectively.

**Table 5:** Association between M-NBI findings and pathological atypia.

M-NBI diagnosis	Pathological cellular atypia		p value
	High-grade n = 22	Low-grade n = 78	
IMVP, mean ± SD (%)	9.0 ± 5.3 (64%)	6.4 ± 4.5 (46%)	0.018
IMSP, mean ± SD (%)	9.3 ± 3.7 (66%)	7.5 ± 3.6 (54%)	0.024

	Pathological Architectural atypia		p value
	High-grade n = 51	Low-grade n = 49	
IMVP, mean ± SD (%)	8.3 ± 4.9 (59%)	5.6 ± 4.3 (40%)	0.003
IMSP, mean ± SD (%)	9.2 ± 3.4 (66%)	6.6 ± 3.4 (47%)	< 0.001

Pathological cellular and architectural atypia were classified as high-grade and low-grade and the mean ± SD (%) of 14 endoscopists who diagnosed IMVP and IMSP were shown.

## Discussion

This is the first study to show that M-NBI reduces underdiagnosis of cancer for matched gastric cancer and gastric adenoma. There are no guidelines for the management of gastric adenomas and they may not always be treated. However, some of these tumors contain cancer, and the accurate diagnosis of cancer, especially in these tumors that are not easily diagnosed by C-WLI or biopsy, is an important clinical issue that has not yet been resolved. In early gastric cancer and gastric adenoma, with C-WLI there are some findings that can be used to diagnose cancer, such as the size of more than 20 mm, depression, and redness. Still, all of these findings have low sensitivity, and a method to reduce the underdiagnosis of cancer is needed^6–8^.

M-NBI is widely used in Japan because it improves cancer diagnosis with a minimal increase in patient burden^16^. There are a few reports stating that M-NBI is effective for differentiating gastric cancer and adenoma. There is a wide range of sensitivity and specificity in previous reports. Some papers show more than 80% sensitivity and specificity, and others show about 40% specificity for differentiation of cancer and adenoma with M-NBI^12,17^. However, no report has focused only on lesions that are difficult to differentiate by C-WLI, which is the problem in clinical practice. Therefore, in this study, we purposely excluded gastric cancers that can be easily diagnosed by C-WLI and examined the effect of M-NBI on gastric adenomas and gastric cancers that are clinically difficult to diagnose.

In pre-matching cases, the mean size of the cancer group was larger than the adenoma group, and the frequencies of red color and depression were significantly higher in the cancer group than in the adenoma group as in previous reports.

Although the diagnostic rate depends on the target lesion type, the mean accuracy of 100 cases diagnosed with C-WLI was as low as 55.9% in post-matching cases, indicating that the accuracy of endoscopic diagnosis using only C-WLI is limited. Even in these difficult to differentiate cases, C-WLI + M-NBI showed significantly higher sensitivity than C-WLI. Thus, M-NBI can effectively diagnose cancer by focusing on IMVP and IMSP, even in cases of matched gastric tumors that are difficult to differentiate by C-WLI alone. For this reason, M-NBI is useful in reducing the underdiagnosis of cancer and resection should be considered when there are M-NBI findings suggestive of cancer, even if C-WLI findings alone are not suggestive of cancer. However, the increase in sensitivity was not high, suggesting that the additional effect of M-NBI is limited.

**Figure 2:**
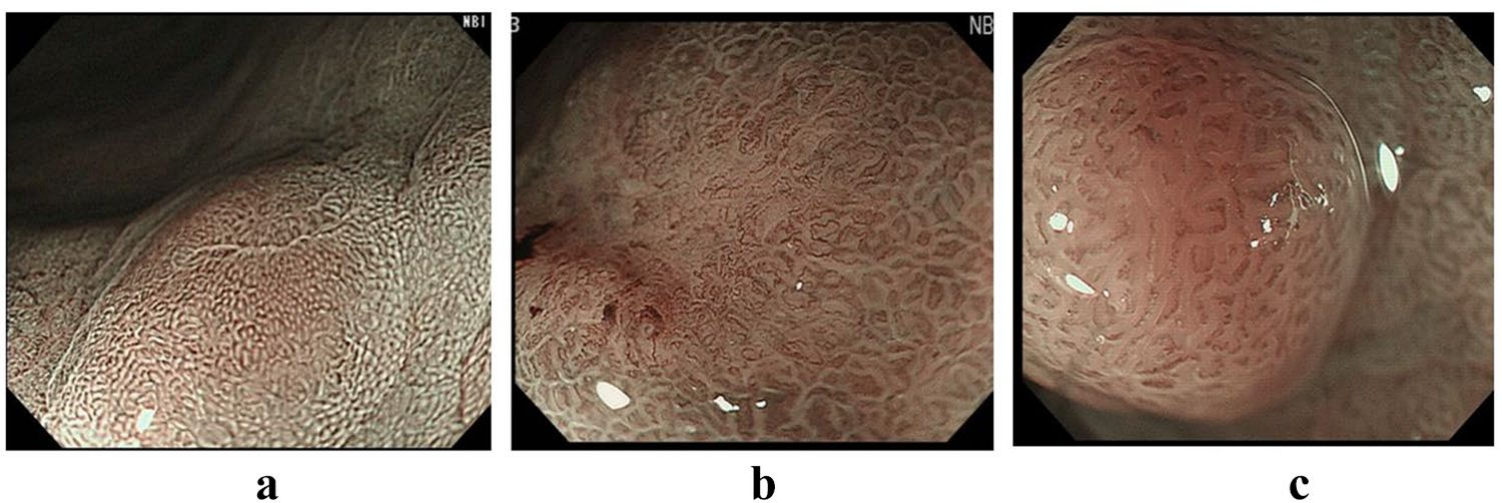
(**a**) Is a representative case of gastric adenoma with 100% accuracy in M-NBI correctly diagnosed by all endoscopists. (**b**) Is a representative case of gastric cancer correctly diagnosed by all endoscopists. (**c**) Is a representative case of gastric cancer correctly diagnosed by only 21% of endoscopists.

As for the inter-endoscopist agreement, Fleiss’s kappa was 0.321, a fair agreement. The percentage of endoscopists whose diagnosis was consistent with the pathology per case ranged from 0 to 100%, suggesting that some cases were easy to diagnose endoscopically, and others were difficult (Fig. 1). There was 1 case of gastric adenoma and 15 cases of gastric cancer which were diagnosed with 100% accuracy using C-WLI + M-NBI (Fig. 2a,b). For the conditions in Fig. 2a, no endoscopists diagnosed either IMVP or IMSP, whereas for Fig. 2b, all endoscopists diagnosed IMVP, and 9 of 14 (64%) endoscopists diagnosed IMSP. It was easy to determine the presence or absence of IMVP and IMSP in these cases, and the diagnostic accuracy was high. In comparison, there were low accuracy cases even with C-WLI + M-NBI (Fig. 2c). In Fig. 2c, only 1 out of 14 endoscopists (0.1%) diagnosed IMVP and only 2 out of 14 endoscopists (0.1%) diagnosed IMSP despite cancer. It may be difficult to diagnose such a lesion by M-NBI alone. In the report that e-learning of M-NBI increased the diagnostic rate of gastric cancer, the fact that e-learning had no effect on raised or flat lesions, which are common in gastric adenomas, also indicates the difficulty of differentiation^18^.

Regarding the long-term follow-up of LGD, Yamada et al. reported that only 1 of 38 cases (3%) were upgraded from category 3 (low-grade adenoma/dysplasia) to category 4 (noninvasive neoplasia, high-grade) by biopsy at a median follow-up of 4.7 years^19^. Rugge et al. reported that 8 of 90 patients (9%) were diagnosed with invasive cancer from LGD at a mean 48 months of follow-up^20^. Okamoto et al. reported that 129 of 376 patients (34%) with gastric adenoma were diagnosed as cancer at the 5-year follow-up, and metachronous gastric cancer was diagnosed in 1.5% of the patients annually^21^. Considering the diagnostic limitations of M-NBI, regular follow-up may be necessary even for patients with endoscopically diagnosed adenoma.

In addition, we were able to show that IMVP and IMSP in M-NBI were associated with pathological high-grade cellular atypia and high-grade architectural atypia respectively. In a long-term follow-up of patients with gastric noninvasive neoplasia for more than 12 months, there was a significant difference in the number of patients diagnosed with invasive carcinoma during the follow-up period: LGD was 9% vs. HGD was 69%^20^. Sakurai et al. reported that subsequent ESD demonstrated submucosal infiltration and/or venous invasion in 3.8% and 1.3% in HGD cases^22^. The pathological diagnosis based on cellular and architectural atypia in Japan may be earlier than by pathologists in the in the West. In this study, IMVP and IMSP were shown to be associated with these cellular and architectural atypia, and attention to the findings of IMVP and IMSP may allow a somewhat earlier diagnosis. This study proves the concept that endoscopic diagnosis can provide a diagnosis that is highly correlated to pathology. In recent years, endoscopes capable of 520 × magnification and evaluation of cell nuclei have been released. However, these endoscopes are still not as versatile as the magnifying endoscope used in this study due to the need for staining and observation techniques. In the future, with further advances in endoscopy, magnifying endoscopy will be closer to the pathological diagnosis, and optical biopsy may become the golden standard for clinical decisions in the future.

This study has several limitations. It was a single-center retrospective study with a limited number of cases. In this study, all images in which the target lesion could be at least partially visualized were extracted to reduce selection bias in endoscopic images, but because diagnosis is made retrospectively with a limited number of images, less information was available than in real-time diagnosis, which may affect the diagnosis. Eradication history was not considered for *H. pylori* infection.

In addition, since gastric adenomas with atypical endoscopic findings are more likely to undergo resection, there is a possibility of selection bias, especially for gastric adenoma cases. A prospective, multicenter study to confirm the results of this study is desirable.

In conclusion, M-NBI is useful in differentiating gastric intramucosal carcinoma from gastric adenoma and can reduce the underdiagnosis of cancer and resection should be considered when there are M-NBI findings suggestive of cancer. This study also shows IMVP and IMSP observed with M-NBI were associated with pathological high-grade cytological and architectural atypia.

## Methods

### Definition of gastric cancer terminology

This is a single center retrospective analysis on endoscopic diagnosis of gastric adenoma and gastric cancer. All resected lesions were histologically diagnosed by pathologists specializing in gastroenterology. Location, macroscopic type, histologic type, and invasion depth were defined according to the Japanese classification of gastric carcinoma by the Japanese Gastric Cancer Association^23^. Vienna classification Category 3 (low-grade dysplasia) corresponds to adenoma in the Japanese classification, and Category 4 and 5 (high-grade neoplasia) to carcinoma.The macroscopic type of early gastric cancer was further classified into 2 groups: depressed (type 0–IIc or type 0–III) or non-depressed (other than type 0–IIc or type 0–III).

Pathological cellular and architectural atypia were reevaluated by two pathologists specializing in gastroenterology.

### Patients

A comprehensive retrospective analysis of each patient’s medical record was performed after approval by the Research Ethics Committee, Graduate School of Medicine and Faculty of Medicine, The University of Tokyo (No. 2058). Written informed consent was waived by the by the Research Ethics Committee, Graduate School of Medicine and Faculty of Medicine, The University of Tokyo owing to the retrospective design of this study. The present study was performed in accordance with the Declaration of Helsinki.

**Figure 3:**
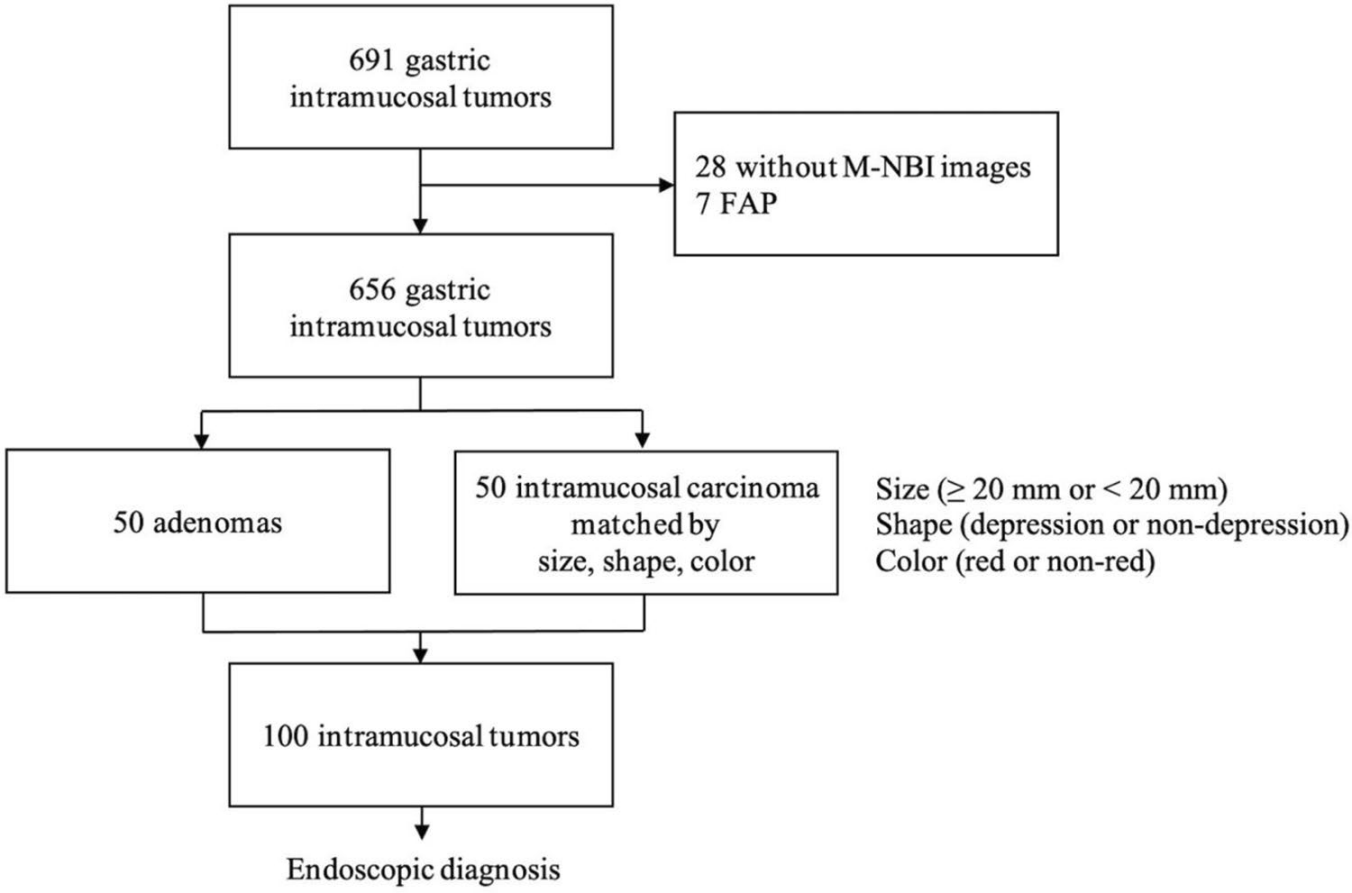
Patient enrollment and extraction. In total, 50 adenomas and 50 intramucosal carcinomas matched by tumor size (≥ 20 mm or < 20 mm), shape (depression or non-depression), and color (red or non-red) were extracted. Endoscopic diagnosis was performed on 100 of these intramucosal tumors.

In total, 691 cases with intramucosal well differentiated adenocarcinoma (tub1) and gastric adenoma diagnosed after endoscopic resection at the University of Tokyo from April 2011 to July 2018 were retrospectively reviewed. The following were excluded: cases without M-NBI images and cases with familial adenomatous polyposis (FAP). After performing 1 to 1 matching by size (≥ 20 mm or < 20 mm), shape (depressed or non-depressed) and color (red or non-red), a total of 50 cases of adenomas and 50 cases of cancers were included for analysis (Fig. 3). If there were multiple candidate lesions, the lesion with the closest ESD date was selected to minimalize possible differences in endoscopic and treatment conditions. *H. pylori* infection status was assessed with either *H. pylori* antibody, urea breath test, stool antigen test, or rapid urease test regardless of *H. pylori* eradication history. Mucosal atrophy was divided into the closed type and open type by the Kimura-Takemoto classification^24^.

### Endoscopy procedures

Patients were prepared with 10,000 units of Pronase and 1 g of sodium bicarbonate, and 40–80 mg of dimethicone before the endoscopic examination to remove mucus. A magnifying upper gastrointestinal endoscope (GIF-Q260Z or GIF-Q290Z, Olympus, Tokyo, Japan) and electronic endoscopy system (EVIS LUCERA or EVIS LUCERA ELITE, Olympus, Tokyo, Japan) were used. A video processor was set up as follows: color mode 1 and structure stressed A mode: level 5 or color mode 1 and structure stressed B mode: level 6 during conventional white light endoscopy; and color mode 1 and structure stressed B mode: level 8 during magnifying endoscopy with NBI. A soft black hood was mounted at the tip of the endoscope to obtain a stable magnification image.

### Endoscopic diagnosis and assessment of M-NBI effect

**Figure 4:**
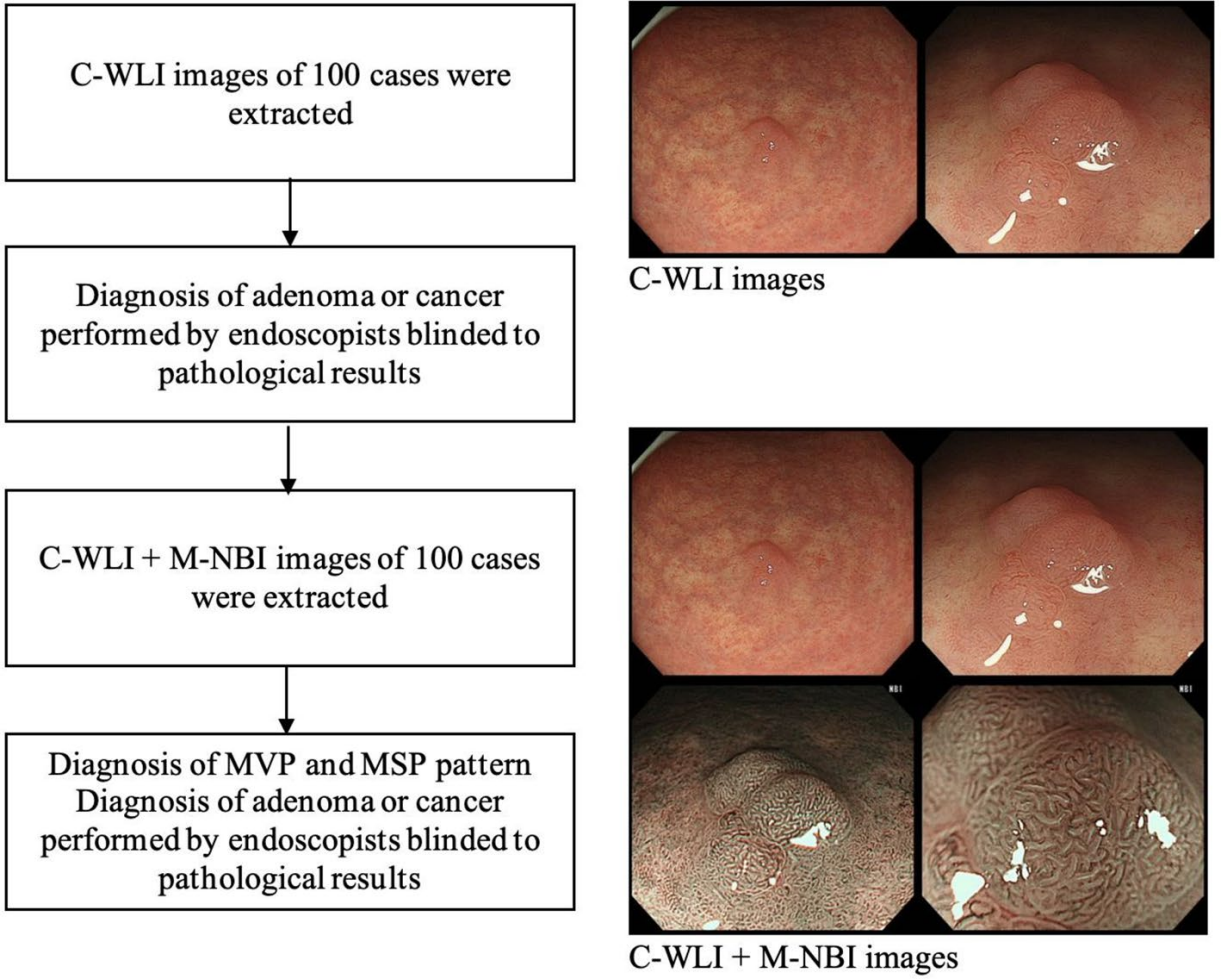
Flow chart of endoscopic diagnosis.

For each of the 50 cancers and 50 adenomas, endoscopic images of the target lesion were extracted from the medical records. All images in which the target lesion could be at least partially visualized were extracted, resulting in a total of at least two images each of C-WLI and M-NBI, including close-up and distant views when possible. Fourteen endoscopists participated in this study. Seven were experienced endoscopists (experts) with more than three years of M-NBI experience and the remaining seven were endoscopic fellows (non-experts) with less than three years of M-NBI experience. All endoscopists performed a differential diagnosis of either gastric cancer or adenoma with only C-WLI. Subsequently, they performed quantitative diagnosis of microvascular and microsurface patterns, and differential diagnosis of adenoma or cancer after observing the C-WLI and corresponding M-NBI images (Fig. 4). For analytical purposes, an endoscopic differential diagnosis of cancer was defined as “positive” and adenoma as “negative” to assess the efficacy of M-NBI by using pathological diagnosis as a golden standard.

For these 100 cases, the sensitivity, specificity, accuracy, positive predictive value (PPV), and negative predictive value (NPV) of each endoscopist were determined. The mean of sensitivity, specificity, accuracy, PPV and NPV of 14 endoscopists with C-WLI was defined as the diagnosis result with C-WLI. In the same way, the mean values of sensitivity, specificity, accuracy, PPV and NPV of 14 endoscopists with C-WLI + M-NBI were determined, and the results of C-WLI and C-WLI + M-NBI were compared. The percentage of an accurate diagnosis for each case was defined as the percentage of endoscopists whose endoscopic diagnosis was consistent with the pathological diagnosis. In addition, the association between diagnostic accuracy and background factors was also investigated.

### Association between M-NBI findings and pathological atypia

One hundred gastric tumors were reevaluated for cytological and architectural atypia by two expert gastrointestinal pathologist (H.A. and T.U.). Cellular atypia was divided into two categories, high-grade and low-grade, based on cytological changes (variably sized and enlarged nuclei, rounded nuclei, loss of polarity, prominent nucleoli). Architectural atypia was also divided into two categories, high-grade and low-grade, based on architectural changes (complex budding or branching of glands, back to back glands)^14^. For M-NBI findings, the number of endoscopists who judged the presence of IMVP and IMSP in each case was determined. Next, the association of pathological high-grade cellular atypia and high-grade architectural atypia with the ratio of endoscopic diagnosis of IMVP and IMSP was evaluated.

### Study outcomes and statistical analyses

The primary aim of this study was to assess the efficacy of M-NBI in differentiating gastric cancer from gastric adenoma with matched characteristics. Categorical variables were analyzed using Pearson’s chi-squared test or Fisher’s exact test, and continuous variables were analyzed using Student’s t-test or the Wilcoxon rank-sum test, as appropriate. Paired t-test or Wilcoxon signed-rank test was used to compare diagnostic rate, as appropriate. Two-sided p-values < 0.05 were considered statistically significant. As a measure of inter-endoscopist agreement, Fleiss’s kappa was used. Values of kappa near zero indicate chance agreement only, while values near the maximum of 1 indicate perfect agreement. Kappa is judged as providing poor agreement if k ≤ 0.20; fair agreement if 0.21 ≤ k ≤ 0 0.40; moderate agreement if 0.41 ≤ k ≤ 0.60; substantial agreement if 0.61 ≤ k ≤ 0.80; and good agreement if k > 0.80^25^. Statistical analysis was performed using JMP Pro version 16 (SAS Institute Inc., Cary, NC) and R statistical package (version 4.1.2, R Foundation for Statistical Computing).

## Data Availability

The datasets generated during and/or analysed during the current study are available from the corresponding author on reasonable request.
